# Case report: Use of penicillin G potassium in poloxamer 407 gel to aid in healing of an equine sublingual abscess

**DOI:** 10.3389/fvets.2022.783753

**Published:** 2022-07-29

**Authors:** Elizabeth A. Larsen, Amy C. Lack, Erica Wassack

**Affiliations:** ^1^Oklahoma State University Veterinary Medical Hospital, Stillwater, OK, United States; ^2^Department of Clinical Sciences, College of Veterinary Medicine, Mississippi State University College of Veterinary Medicine, Mississippi State, MS, United States; ^3^Ontario Veterinary College, University of Guelph, Guelph, ON, Canada

**Keywords:** horse, equine, poloxamer 407, wound, abscess

## Abstract

The use of poloxamer 407 gels have been reported in several studies to prolong the release of drugs at the injection site. Oral lesions unrelated to dental disease are rare but may result in ulceration and sequestration of bone. To date, there have been no reports on the use of penicillin G potassium poloxamer 407 gel and its effect on wound healing. The present case report describes the use of a penicillin G potassium poloxamer 407 gel for the treatment of a sublingual abscess involving the mandible in a 20 year old Arabian mare who initially presented with acute onset of dysphagia, hypersalivation, and a mass under the tongue. A presumptive diagnosis of lingual cellulitis was made, and a sublingual abscess ruptured on day 7 of hospitalization. In this case, poloxamer 407 gel was used to decrease wound contamination, protect the exposed mandible, and potentially prolong the release of penicillin G potassium into the wound.

## Introduction

Poloxamers are non-ionic polyoxyethylene–polyoxypropilene–polyoxiethylene tri-block copolymers with many pharmaceutical drug delivery applications (1–4). Polymers such as poloxamer 407 (P407) are able to exhibit sol-to-gel transitions when they are applied at body temperature (5). P407 is the most widely used copolymer (1). Solutions of P407 show in-situ thermoreversible gelation behavior, which permit them to be stored and administered in cold liquid form (1, 3, 4). P407 cold solution forms a gel in situ when administered at the site of injection and heated (1, 3, 4). The temperature needed to solidify the gel is dependent upon the concentration of P407 (4). The P407 gel is biocompatible with cells and body fluids (1–3).

The use of P407 gels have been reported in several studies to prolong the release of drugs at the injection site, however hydrogels such as P407 have been limited to carrying hydrophilic drugs rather than hydrophobic drugs due to the limited quantity and homogeneity of loaded hydrophobic drugs in hydrogel matrices (6, 7). A 2005 study by Ricci et.al showed that the use of P407 gels prolongs the residence time of lidocaine at the injection site, sustains drug release and increases therapeutic efficacy (1). Additionally, the combination of vancomycin with P407 allowed for prolonged release of the time-dependent antibiotic *in vitro* and *in vivo* (8). It has also been shown that subcutaneous carboplatin in P407 can be used *in vivo* providing direct tissue exposure to carboplatin without significant local effects, systemic absorption, or wound healing complications (9). Furthermore, a study on the use of poloxamer-based binary hydrogels for delivering tramadol hydrochloride *via* subcutaneous injection showed reduced cytotoxicity, genotoxicity, and prolonged analgesic effects of >72 h (10). Poloxamer 407 has also been used to formulate sublingual or oral preparations, such as sublingual piroxicam and oral doxycycline hyclate (2, 11, 12).

To date, there have been no reports on the use of penicillin G potassium formulated in P407 gel and its effect on wound healing. The present case report describes the use of a penicillin G potassium P407 25% gel for the treatment of a sublingual abscess involving the mandible in a 20 year old Arabian mare.

## Background

A 20 year old Arabian mare presented to Mississippi State University Animal Health Center (MSU-AHC) for acute onset of dysphagia, hypersalivation, and a possible mass under the tongue. There was one other horse on the property who was unaffected. The horse had been vaccinated for eastern and western encephalomyelitis, tetanus, rabies, and west Nile virus six months prior to presentation. The horse had previously been diagnosed with pituitary pars intermedia dysfunction and was being maintained on pergolide 2.2 μg/kg PO every 24 h.

Upon presentation, the horse was bright, alert, and responsive. She weighed 406 kg and had a body condition score of 4/9. Initial physical examination performed revealed a heart rate (40 beats/min), respiratory rate (18 breaths/min) and rectal temperature (38.1°C) were within normal limits. She had moderately increased digital pulses and abnormal growth rings on both front hooves. The submandibular lymph nodes were moderately enlarged and accompanied by moderate facial edema extending from mid-facial crest rostral. The tongue was protruding out of the right side of her mouth, was flaccid, and unable to be retracted into the oral cavity. On oral exam a necrotic mass was present at the base of the tongue incorporating the frenulum of the tongue and left sublingual tissue. The remainder of the mouth was normal. A brief neurological exam noted bilateral sensory deficits rostral to the infraorbital canal and lower lip paralysis. The remainder of the physical exam was within normal limits. A venous blood sample was submitted for complete blood count and serum chemistry. Complete blood count revealed a moderate leukocytosis 16.54 10^9^/L [reference range (rr) 5.0–11.9 × 10^9^/L] and neutrophilia 15.382 10^9^/L (rr 2–6 × 10^9^/L), mild monocytosis 992/μL (rr 0–800/μL), and severe lymphopenia 165/μL (rr 1,250–5,000/μL). Serum chemistry revealed a mild hyperproteinemia 9.2 g/dl (rr 6.1–8.4 g/dl), mild hyperglobulinemia 5.2 g/dl (rr 2.5–4.0 g/dl), and moderate hyperglycemia 218 mg/dl (rr 60–122 mg/dl). A presumptive diagnosis of lingual cellulitis with cranial nerve VII and V deficits was made.

### Treatment

An intravenous (IV) catheter was placed in the left jugular vein, and the horse was started on broad spectrum antimicrobials using enrofloxacin 6.6 mg/kg IV every 24 h and potassium penicillin 22,000 IU/kg IV every 6 h, along with flunixin meglumine 1.1 mg/kg IV every 12 h, and was continued on the previously prescribed pergolide 2.2 μg/kg PO every 24 h. The horse also received 5 L of fluids as a precautionary measure, as her ability to drink was unknown. After being observed drinking water, the fluids were discontinued.

Skull radiographs were performed, which revealed a smoothly marginated, soft tissue opaque, ovoid mass with moderate amounts of heterogenous gas centrally that was superimposed over the body of the tongue measuring ~8.7 × 3.9 cm, and another smoothly marginated, soft tissue opaque, semi-circular mass with a moderate amount of heterogenous gas centrally which overlapped and was rostral to the previously described mass measuring ~4.4 × 1.4 cm. These masses were interpreted as an abscess or necrotic neoplasm.

On day 3 of hospitalization, a firm swelling of the rostral intermandibular space was observed, and an ultrasound was performed. There were several areas of subcutaneous gas noted in the rostral intermandibular region, which was suggestive of either a developing abscess or a previous gas tract from a penetrating wound and associated cellulitis, but overall ultrasound was inconclusive. No foreign material was observed by ultrasound. This finding was consistent with the findings of the skull radiographs. It was decided to observe the swelling and re-ultrasound at a later date.

On day 4 of hospitalization, a repeat complete blood count and serum chemistry were performed. Complete blood count revealed a resolving neutrophilia 7.056 10^9^/L (rr 2–6 × 10^9^/L), and lymphopenia 537/μL (rr 1,250–5,000/μL). Although not out of reference range, the fibrinogen had increased to 500 mg/dl (rr 100–500 mg/dl). Serum chemistry revealed a resolved hyperglycemia and improved hyperproteinemia 8.5 g/dl (rr 6.1–8.4 g/dl).

On day 7 of hospitalization, the intermandibular swelling was ultrasonographically examined. There were numerous areas of subcutaneous gas noted in the rostral intermandibular region, and areas of fluid pocketing. The previously homogenously firm area now had a soft pocket. The area was determined to be an abscess, which was then lanced on the ventral aspect of the swelling *via* a stab incision through the skin. Approximately 40 ml of purulent material was drained. The abscess was flushed with sterile saline, and left to heal by second intention. Sedated oral exam revealed that the intra-oral abscess had ruptured, and there was now a cavity at the base of the tongue, measuring ~1.5 cm in diameter and ~7 cm deep. The cavity was flushed using a dilute chlorohexidine solution and debrided with a curette. The medial aspect of the left mandible was exposed within the wound cavity, with no intact periosteum visible. Rinsing the mouth with a dilute chlorohexidine solution of 0.2% was added to the treatment protocol every 6 h to decrease feed packing into the wound.

On day 8 of hospitalization, the wound was filled with large amounts of feed material. It was flushed with sterile saline, and a mixture of manuka honey, metronidazole, and cephaperin benzathine were attempted to be applied to the open wound in the oral cavity. However, the salve immediately ran out when the horse's head was lowered. The intermandibular abscess was flushed using sterile saline.

**Figure 1:**
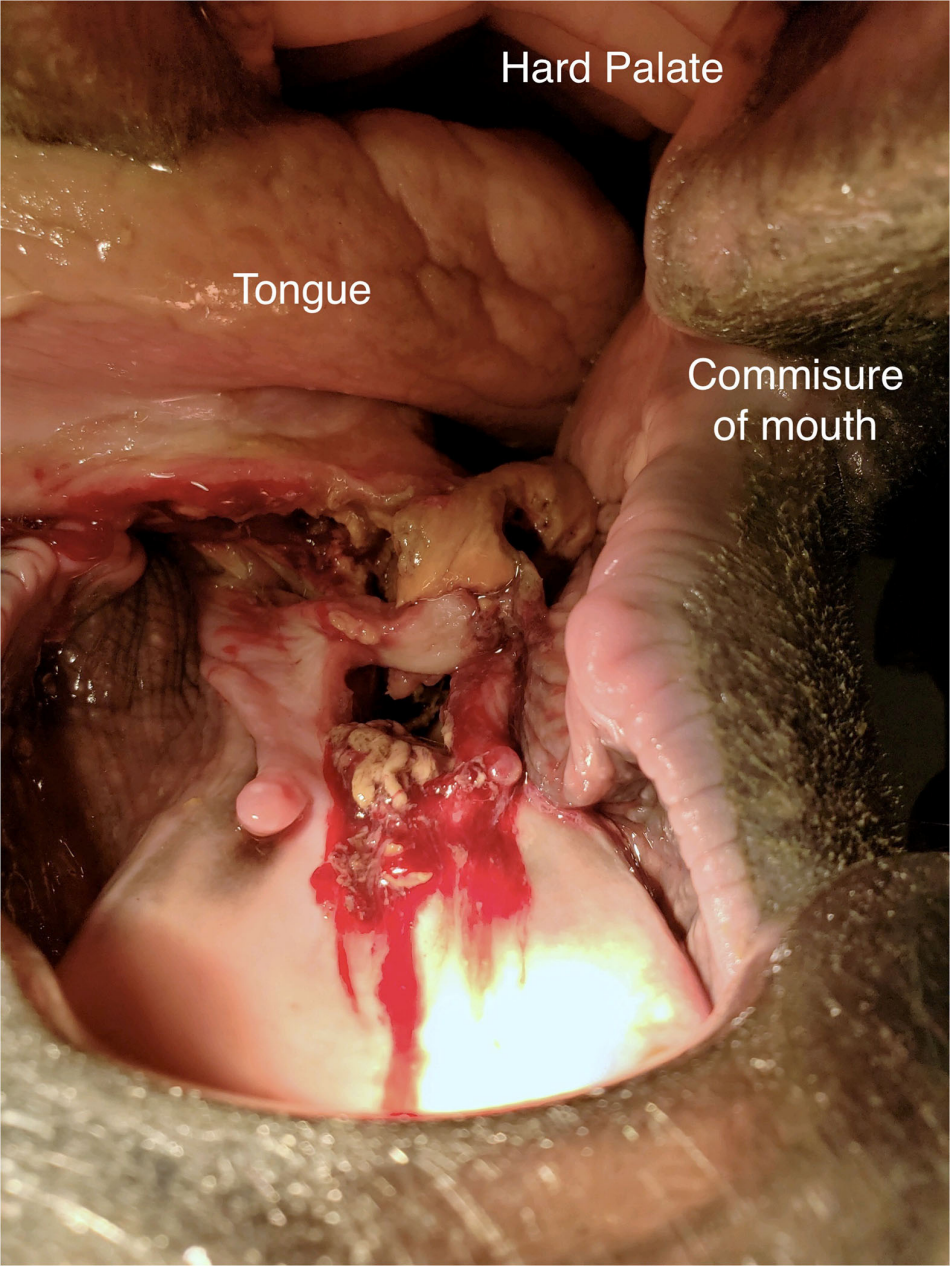
Image of the intra-oral lesion on day 9 (treatment day 0) after initial presentation

On day 9 of hospitalization (Figure 1), a penicillin G potassium P407 25% gel (PGKPG) at a strength of 200,000 units/ml was compounded. Penicillin G potassium was chosen due to its broad-spectrum bactericidal activity and for the potential of continuing the previously implemented intravenously penicillin G potassium. P407 25% gel base was compounded using 12.5 g of P407 and 33 ml of sterile water, which created a total volume of 50 ml of P407 gel at a concentration of 25%. This concentration of P407gel was chosen as to decrease the amount of time needed until formation of solid like gel during application. The 25% gel was then autoclaved to kill any microorganisms and spores for sterilization. Penicillin G Potassium 20 MU was reconstituted using 11.5 ml of sterile water for injection, to create a concentration of 1,000,000 units/ml. Four mililiter of reconstituted penicillin G potassium and 16 ml of 25% P407 gel were combined using syringe-to-syringe method to create a total volume of 20 ml of PGKPG. The compounded product was stored in the refrigerator until administration to retain liquid state.

The horse was sedated and an oral speculum was placed. The mouth and wound cavity were flushed using 0.2% chlorhexidine solution. The horse's head was then held above wither height, and the wound was dried using dry gauze. The wound cavity was then filled with 20 ml of PGKPG, and the horse's head was held above the withers until PGKPG solidified and set in the wound. The head was lowered, and the gel was observed to remain in the wound.

### Results

**Figure 2:**
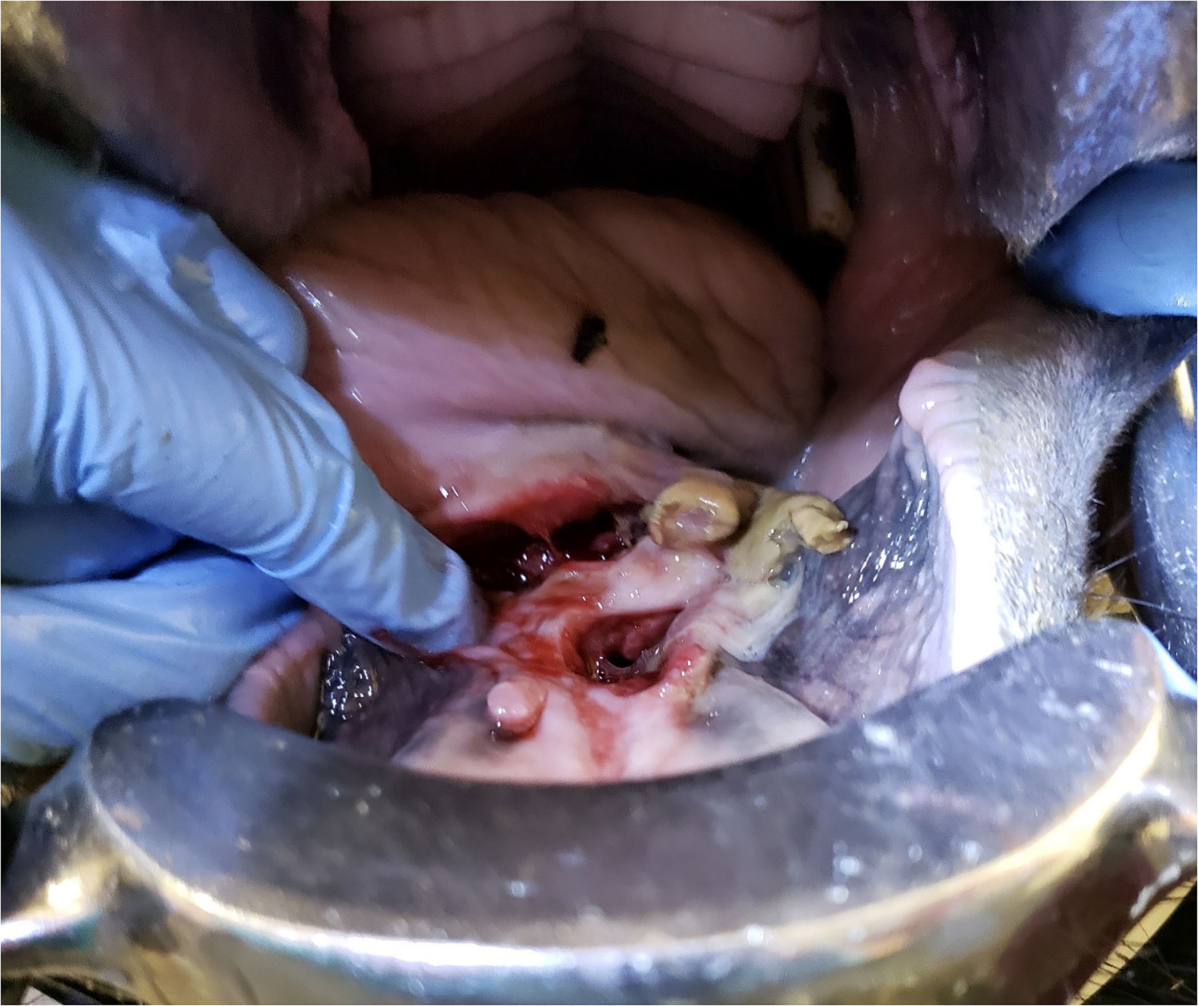
Image of the intra-oral lesion on day 11 (treatment day 2) after initial presentation

**Figure 3:**
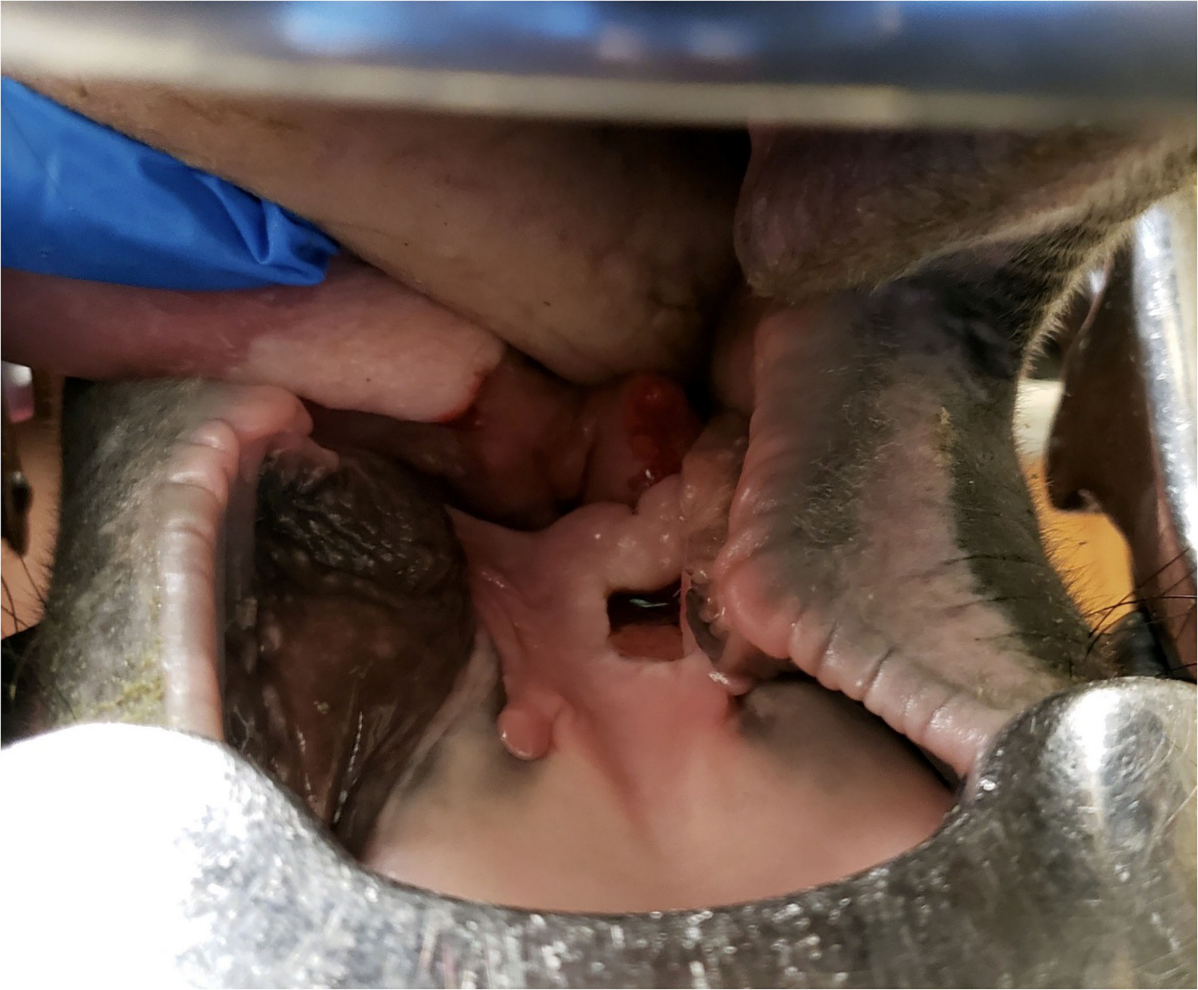
Image of the intra-oral lesion on day 23 (treatment day 14) after initial presentation

Treatment with PGKPG was repeated on days 10, 11 (Figure 2), 14, 16, 17, 19, 21, and 23 (Figure 3) of hospitalization. It was observed that there was PGKPG still coating the interior of the abscess wound at 1- and 2-days post administration, but not at 3 days. During that time, the horse regained use of her tongue and was gradually transitioned from a liquid mash to a solid mash diet consisting of alfalfa pellets, senior grain, and omalene-100, along with one flake of hay per day. The systemic intravenous antibiotics (enrofloxacin and penicillin G potassium) were discontinued on day 10 of hospitalization after it was observed that PGKPG still coated the interior cavity of the wound. The horse continued to receive flunixin meglumine IV twice daily intravenously until day 21, when the intravenous catheter was removed. The horse was then started on oral flunixin meglumine paste once daily. On day 23 of hospitalization, repeat skull radiographs were performed, which showed no evidence of osteomyelitis of the left mandible. There was partial resolution of the previously described soft tissue and gas opaque structures superimposed over the body of the tongue. A final treatment of intralesional PGKPG was performed.

On day 25 of hospitalization, the horse was discharged into her owner's care. At this time the sublingual wound was 0.75 cm in diameter and 3 cm deep, and previously described neurologic deficits had resolved. Discharge instructions were to administer flunixin meglumine paste orally every 24 h, pergolide orally every 24 h, and to rinse the mouth with water twice daily. The referring veterinarian performed a recheck oral examination 1 week after discharge and reported continued wound healing. The horse presented for a recheck 63 days after initial presentation, which showed the wound to be nearly filled with granulation tissue. Mandibular radiographs showed no evidence of sequestrum formation.

## Discussion

This case report highlights the role of antibiotic infused P407 gel in wound healing. To the authors' knowledge, there are no previous reports of using PGKPG to aid in the healing of wounds. In this case, the P407 gel was used to decrease wound contamination, protect exposed mandible, and potentially prolong release of penicillin G potassium into the wound.

Hudson et al. (13) describes that the numerous causes of dysphagia in horses can be classified as painful, obstructive or neurogenic, and the list of possible differential diagnoses can include: dental problems, oral lesions, head trauma, strangles, foreign bodies, neoplasia, guttural pouch disease, pharyngeal paralysis, lead poisoning, botulism, hepatoencephalopathy, grass sickness, viral encephalomyelitis, equine protozoal myeloencephalitis, nigropallidal encephalomalacia, choke and esophageal stricture. Oral lesions unrelated to dental disease are considered to be rare. In a 2010 survey of 556 horses presenting to a Canadian slaughter house, oral abscesses and masses were observed in 3.6 % of the population (14). In some instances of oral wounds, debridement and lavage followed by primary closure is indicated, although most wounds are allowed to heal by second intention because the oral cavity has an excellent blood supply and heals rapidly (15). However, examination of this region may reveal ulceration and, in some cases, sequestration of bone (15).

There was no initial evidence of osteomyelitis of the exposed mandible at 13 and 63 days after initial exposure, despite the prolonged period of time of exposed bone. It is possible that the use of anti-microbial impregnated P407 gel on exposed bone may help prevent the development of osteomyelitis. A 2017 study reported that the infection rate is 5%−15% in fracture fixation devices and 0.3%−5% in joint prosthesis in humans in United States of America (16). Implant associated infection after tibial plateau leveling osteotomy in dogs occurs in ~7.3%−7.4% of cases (17, 18). Systemic penicillin and gentamicin in combination are commonly used to treat osteomyelitis in horses until definitive culture and sensitivity results are known (19). The use of antimicrobial-impregnated polymethylmethacrylate (AIPMMA) implants for prevention and treatment of osteomyelitis in horses has drastically improved the success rate (19). Common complications of AIPMMA implants have been soft tissue damage during removal and the formation of fibrous connective tissue complicating removal (19). Additionally, AIPMMA should not be placed in joints due to its abrasive nature on cartilage (19, 20). It is possible that use of P407 Gel on exposed bone and implants prior to closure, and within joints, could provide prolonged anti-microbial prophylaxis, decreasing the development of osteomyelitis post operatively. Furthermore, a study using isolated autologous chondrocytes, polyglycolic acid, and P407 gel successfully *in vivo*-engineered hyaline cartilage and repaired articular cartilage defects, showing that P407 gel is safe for use in joints (21, 22). Further investigation of this topic is warranted.

While this study did not use nanoparticles, the use of nanosystems within poloxamer hydrogels has much promise in modern medicine. Cristiano et al. (23) demonstrated the topical application of vesicular nanocarriers embedded in poloxamer hydrogels for percutaneous drug delivery allows for the passage of active ingredients which are normally not able to cross the stratum corneum and allows a more controlled release of the drug. Nanoparticles within poloxamer hydrogels have also been used to deliver specific and controlled release of clodronate in joints to reduce degeneration associated with rheumatoid arthritis (24). Finally, a 2021 study by Liu et al. (21) used silver nanoparticles within poloxamer gels as a carrier in order to break down bacterial biofilms and eliminate *Enterococcus faecalis* in root canal therapy.

Limitations of this case report include a lack of comparative studies between PGKPG and free penicillin G. The ability and length of time of release of penicillin G from PGKPG was also not evaluated, however the horse was discontinued from systemic antimicrobials on day 10 of hospitalization and the wound did not regress while using the PGKPG despite constant contamination from the oral cavity. These limitations require further evaluation for the use of PGKPG in other clinical settings.

## Concluding remarks

In conclusion, PGKPG appeared safe and, in this case, beneficial for healing a highly contaminated wound. Although the exact duration of time in which the gel remained in contact with the oral mucosa was not evaluated in this case report, the authors' noticed the wound to be coated with PGKPG at 1- and 2-days post application, and a marked improvement in gross wound contamination after applications. The authors' believe that PGPKP gel should be considered as an adjunctive therapeutic for contaminated wounds. Further investigation of antimicrobial infused P407 gel for use in wounds and surgical sites in horses is warranted.

## Data availability statement

The original contributions presented in the study are included in the article/supplementary material, further inquiries can be directed to the corresponding author.

## Ethics statement

Ethical review and approval was not required for the animal study because Permission was obtained from the owner of the horse described in this case study for publication of the case for educational purposess. Written informed consent was obtained from the owners for the participation of their animals in this study.

## Author contributions

The horse described in this case was referred to Mississippi State University Animal Health Center where the case was primarily managed by AL and EL. Pharmacologic advising and compounding was performed by EW. Contributions for preparation of the manuscript were made by EL, AL, and EW. All authors contributed to the article and approved the submitted version.

## Conflict of interest

The authors declare that the research was conducted in the absence of any commercial or financial relationships that could be construed as a potential conflict of interest.

## Publisher's note

All claims expressed in this article are solely those of the authors and do not necessarily represent those of their affiliated organizations, or those of the publisher, the editors and the reviewers. Any product that may be evaluated in this article, or claim that may be made by its manufacturer, is not guaranteed or endorsed by the publisher.
